# Assessing Large Language Models in Building a Structured Dataset From AskDocs Subreddit Data: Methodological Study

**DOI:** 10.2196/74094

**Published:** 2025-10-22

**Authors:** Quinn Snell, Chase Westhoff, John Westhoff, Ethan Low, Carl L Hanson, E Shannon Neeley Tass

**Affiliations:** 1 Brigham Young University, 3361 TMCB, Provo, UT, 84602, United States, 1 8014225098; 2 University of Nevada, Reno, Reno, NV, United States

**Keywords:** large language models, artificial intelligence, Reddit, unstructured text analysis, data extraction

## Abstract

**Background:**

In an era marked by a growing reliance on digital platforms for health care consultation, the subreddit r/AskDocs has emerged as a pivotal forum. However, the vast, unstructured nature of forum data presents a formidable challenge; the extraction and meaningful analysis of such data require advanced tools that can navigate the complexities of language and context inherent in user-generated content. The emergence of large language models (LLMs) offers new tools for the extraction of health-related content from unstructured text found in social media platforms such as Reddit.

**Objective:**

This methodological study aimed to evaluate the use of LLMs to systematically transform the rich, unstructured textual data from the AskDocs subreddit into a structured dataset, an approach that aligns more closely with human cognitive processes than traditional data extraction methods.

**Methods:**

Human annotators and LLMs were used to extract data from 2800 randomly sampled r/AskDocs subreddit posts. For human annotation, at least 2 medical students extracted demographic information, type of inquiry (diagnosis, symptom, or treatment), proxy relationship, chronic condition, health care consultation status, and primary focus topic. For LLM data extraction, specially engineered prompts were created using JavaScript Object Notation and few-shot prompting. Prompts were used to query several state-of-the-art LLMs (eg, Llama 3, Genna, and GPT). Cohen κ was calculated across all human annotators, with this dataset serving as the gold standard for comparison with LLM data extraction. A high degree of human annotator reliability was observed for the coding of demographic information. Lower reliability was seen in coding the health-related content of the posts.

**Results:**

The highest performance scores compared with the gold standard were achieved by Llama 3 70B with 7 few-shot prompt examples (average accuracy=87.4) and GPT-4 with 2 few-shot prompt examples (average accuracy=87.4). Llama 3 70B excelled in coding health-related content while GPT-4 performed better coding demographic content from unstructured posts.

**Conclusions:**

LLMs performed comparably with human annotators in extracting demographic and health-related information from the AskDocs subreddit unstructured posts. This study validates the use of LLMs for analyzing digital health care communications and highlights their potential as reliable tools for understanding online behaviors and interactions, shifting toward more sophisticated methodologies in digital research and practice.

## Introduction

### Background

The advancement of digital health care, especially highlighted during the COVID-19 pandemic, has significantly increased the reliance on online platforms for medical consultation and advice, profoundly changing how individuals seek medical advice over the last 2 decades [1,2]. With a growing focus on platforms such as Reddit for “Ask the Doctor” services, Reddit’s r/AskDocs subreddit has become a vital forum for such interactions, growing to more than 550,000 subscribers by January 2024 [3]. This increase, marked by a surge in user engagement starting in 2018, exemplifies the evolving role of social media as a trusted source for medical advice on the web. Recent studies on social media platforms such as Reddit have highlighted active user engagement in health-related discussions, such as medication abortion [4] and dermatology [5]. The r/AskDocs subreddit has been a focal point for analyzing user demographics and health topic trends, marked by a dramatic increase in user posts over time [3]. This trend toward asynchronous health care, where individuals engage with health care professionals and peers over digital platforms, underscores a shift in how medical advice is sought and dispensed in the modern era.

The potential to harness insights from these forums is immense, offering a unique window into patient concerns, misconceptions, and the public’s health-seeking behaviors. However, the vast, unstructured nature of forum data presents a formidable challenge; the extraction and meaningful analysis of such data require advanced tools that can navigate the complexities of language and context inherent in user-generated content. Recently, large language models (LLMs) have been used to extract data from unstructured text. These models can navigate much of the nuances of the languages in forums and social media to extract usable data at what appears to be similar to human levels. Understanding the strengths and the limitations of the use of LLMs as compared with humans for data extraction is the focus of this research.

### Traditional Methods of Information Extraction From Text

Regular expressions (often shortened to regex), a staple in text processing, and many other traditional natural language processing tools offer rule-based approaches to identifying specific patterns within text. For example, regex can be used to locate all instances of email addresses or phone numbers within a database by defining the patterns that match email addresses and phone numbers. In the realm of data extraction from web-based health forums such as the AskDocs subreddit, the intricacy of human language and the unstructured nature of user submissions present significant challenges. Users frequently provide a wealth of information, albeit in varied formats that defy simple pattern matching. The diversity in the presentation of these data complicates the task of developing regular expressions that can accurately and consistently extract the desired information [6].

To illustrate the complexity of this task, consider the following examples that represent patterns seen in posts on the AskDocs subreddit (Table 1). These examples highlight the variability and nuanced nature of the information provided by users, underscoring the difficulties in crafting regex patterns capable of effectively parsing and categorizing these data.

**Table 1.** Examples of AskDocs subreddit user submissions and their complexity.

Text	Explanation
“I’m worried about a white lump on my elbow. Age: 31; Race: Japanese; Sex: Male”	The text clearly states their age and race but uses “white” first in a medical context, not as a race.
“Mid-30s F here, experiencing severe headaches. Also, I’m 5 foot 6” and around 60-ish kg.”	Use of approximate age (“Mid-30s”) and nonstandard expressions for measurements (“5 foot 6” and “60-ish”).
“I’m a minor, dealing with severe migraines especially during my period. 150 cm, 60 kg”	The text implies the user’s sex through the mention of a menstrual cycle but does not explicitly state it.

These examples underscore the inherent challenges in using regular expressions for data extraction from AskDocs subreddit. The variability in how users report their demographic information, combined with the use of language in medical contexts, necessitates a highly sophisticated system using a variety of regex patterns. Building such a system is not only daunting but also entirely impractical.

### Large Language Models

In contrast, artificial intelligence (AI) and particularly LLMs introduce a paradigm shift in data extraction. LLMs trained on vast corpora of text demonstrate an understanding of language nuances, contextual meaning, and the implicit cues embedded within text. This enables LLMs to interpret and categorize complex information without the need for explicitly defined rules, as is the case with regex [7].

The integration of LLMs in analyzing web-based health forums represents a significant advancement in this field. LLMs trained on extensive datasets have proven effective in understanding and generating human-like text. For instance, the GatorTron model, a large clinical transformer model, demonstrated remarkable performance in extracting and using patient information from clinical narratives [8]. Furthermore, the effectiveness of LLMs in preserving privacy while extracting information highlights their growing importance in sensitive domains such as health care [9].

In addition to their general capabilities, LLMs have shown proficiency in tasks such as information extraction, categorizing text data, and identifying sentiment in complex and unstructured data settings [10]. Another study showed how fine-tuning LLMs such as GPT-3 can accurately extract complex scientific knowledge [11]. This makes them highly suitable for extracting nuanced information from health-related discussions on the web. In summary, the existing research lays a comprehensive foundation for understanding web-based health-seeking behavior, with LLMs playing a crucial role in advancing the understanding and analysis capabilities in digital health communication.

### Research Question and Aim

Amidst this backdrop, this study evaluates the methodology of using LLMs to transform the unstructured, information-rich text data from sources such as the AskDocs subreddit into a structured dataset. Unlike traditional data extraction methods, which struggle with the variability and complexity of natural language, LLMs offer a context-aware, nuanced approach that more closely aligns with human cognitive processes. The following research question seeks to evaluate the methodology’s effectiveness and explore its broader applications: How does the accuracy and agreement of LLMs in labeling web-based health communication compare with human annotators in extracting and categorizing complex information from social media datasets such as the AskDocs subreddit?

This study focuses on the methodology and its validation, aiming not only to demonstrate the feasibility of using LLMs for analyzing web-based health communication but also to explore their broader implications for digital research and practice. To demonstrate the method’s use, a brief analysis of the extracted dataset was conducted to illustrate future use cases. This approach seeks to uncover new avenues for understanding web-based behaviors and interactions.

While this research focuses on extracting structured datasets from health forum data, the applicability of LLMs extends far beyond this realm. Their versatility and advanced understanding of natural language may make them suitable for various fields requiring data extraction and analysis. These fields could include legal document analysis [12], financial report summarization, and sentiment analysis in social media [13-15]. LLMs offer a powerful tool for transforming unstructured text into actionable insights. This broad applicability underscores the transformative potential of LLMs across multiple sectors, promising to revolutionize data analysis and knowledge extraction in an array of disciplines.

## Methods

**Figure 1.**
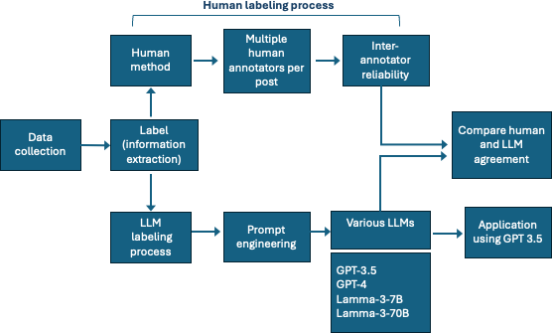
Flowchart of methods used for this study. LLM: large language model.

This section outlines the processes for data collection, human and LLM labeling, and comparison of label similarity between human annotators and LLMs, as outlined in Figure 1. The following sections describe each component of the flowchart in detail.

### Data Collection

The AskDocs subreddit is part of Reddit, which hosts more than 13 billion posts across more than 100,000 subreddits, engaging more than 50 million daily users [16]. Due to limitations in Reddit’s official application programming interface (API) (PRAW) for retrieving historical data, the Pushshift API [17], a third-party archive of Reddit’s post metadata, was used. Despite known gaps in Pushshift’s data coverage, it remains a valuable tool for accessing large volumes of Reddit data, including the AskDocs subreddit.

Data were extracted from AskDocs subreddit posts spanning from inception in July 2013 to October 2022, comprising 1,016,229 posts and 2,122,081 comments. While acknowledging the challenges in obtaining a complete dataset, the available data were substantial for extracting insights and addressing the research objectives of this study. A random sample of 2800 AskDocs subreddit posts was used for comparing data extraction using human labeling and LLM labeling. A different sample of approximately 30,000 AskDocs subreddit posts was used to demonstrate the LLM data retrieval methodology.

### Human-Labeling Process

Human labeling for extracting and categorizing information from AskDocs subreddit posts was essential for this study for multiple reasons. First, it provided a method for creating the “gold standard” dataset used for evaluating the accuracy and reliability of LLMs relative to human-level understanding and judgment. Second, the comparison of LLM annotation against the gold standard, and of individual human annotators against the gold standard, sheds light on the potential of LLMs to augment human efforts in terms of efficiency, scalability, and consistency. Understanding where LLMs excel or fall short compared with human annotators allows for better utilization of capabilities, identification of areas for improvement, and refinement of methodologies to enhance their performance in real-world applications.

In collaboration with the University of Nevada, Reno Medical School, 27 medical students served as human annotators for data labeling. The students were tasked with categorizing the posts according to the extraction criteria outlined in Table 2, which had been reviewed and approved by a physician consultant (JW) to ensure clinical relevance and accuracy. Each medical student was required to read the training material, which outlined the task, explained all the data fields and value options, and had several real Reddit post examples with justifications for each chosen label for that post. The training document, found in Multimedia Appendix 1, was designed to address the various labeling challenges annotators might encounter. These labeling guidelines were followed by each annotator and specifically referred to when resolving differences. While some labeling categories are inherently subjective, they were specifically included to distinguish the kinds of questions users were asking and shed light on the capabilities of LLMs for extracting subjective data.

**Table 2.** Information fields and possible responses for human and large language model data extraction from the AskDocs subreddit posts.

Field	Options
Biological Sex	M, F, Unknown, N/A^a^
Gender Identity	M, F, Other, N/A
Age	A numerical value, Unknown, N/A
Height	Height in formats such as 6’0” or 170 cm, Unknown, N/A
Height Units	Feet/in, cm, m, N/A
Weight	A numerical value, Unknown, N/A
Weight Units	lbs, kg, N/A
Race	White, Asian, Black, Hispanic, Other, Unknown
Diagnosis-Based Medical Inquiry	True, False, N/A
Symptom-Based Medical Inquiry	True, False, N/A
Treatment-Based Medical Inquiry	True, False, N/A
Proxy Relationship	N/A, significant other, friend, child, other
Chronic Condition	True, False, N/A
Healthcare Consultation Status	Preconsultation, in consultation, postconsultation, N/A
Primary Focus Topic	Multiple possible topics

a N/A: not available or not applicable.

The sample size was based on each medical student’s 10-hour time availability. It was assumed that each student annotator could categorize the extraction fields for 1 AskDocs subreddit post in an average of 1.5 minutes, which would yield 400 labeled posts over a 10-hour period. The research team then randomly selected 3600 posts and organized them into 9 batches of 400 posts each to provide a comprehensive snapshot of the prevalent dialogues on the platform. Each batch was then reviewed by 3 medical student annotators to ensure thorough examination of each post from multiple perspectives.

The execution of this plan encountered practical challenges. Despite initial aspirations, only 2 of the 9 batches saw the completion of labeling by all 3 assigned annotators. Five batches had the contribution of 2 students; unfortunately, 2 batches were labeled by a single student. To maintain quality, the team ensured that each post was reviewed by at least 2 individuals to guarantee robustness and reliability of the human-labeled dataset; the decision was made to exclude the batches that were reviewed only by 1 student. As a result, the final dataset comprised 2800 posts, each labeled by at least 2 medical students.

The gold standard dataset was based on the majority response from the human annotations. In cases of disagreement, the lead researcher—who created the labeling guidelines and followed them closely—resolved any disagreement using the same criteria provided in the annotator guidelines. This approach ensured consistency in applying the guidelines across all labeled data. This dataset then served as the gold standard for a comprehensive comparison with the data extraction by various LLMs.

### Human Interannotator Agreement

Cohen κ score, a statistical measure for evaluating the level of agreement between 2 or more annotators, was used to assess interannotator reliability [18]. This measure accounts for the possibility of chance agreement in its calculation, making it a more robust indicator of interannotator reliability than simple percentage agreement. The magnitude of the Cohen κ score indicates the level of agreement, with a score of 1 corresponding to perfect agreement. There are different ways to categorize agreement level using the Cohen κ score. One such categorization proposed by McHugh [19] is shown in Table 3.

**Table 3.** Interpretation of Cohen κ as proposed by McHugh [19].

κ Score	Agreement level
0‐0.2	Almost none
0.21‐0.39	Minimal
0.40‐0.59	Weak
0.60‐0.79	Moderate
0.80‐0.90	Strong
Above 0.90	Almost perfect

In the realm of data labeling, particularly for qualitative data with subjective categories, the Cohen κ score is a standard metric for measuring annotator consistency. Interannotator agreement with a Cohen κ score is illustrated in the matrix, showcasing the degree of consensus among different pairs of human annotators on various categories.

**Figure 2.**
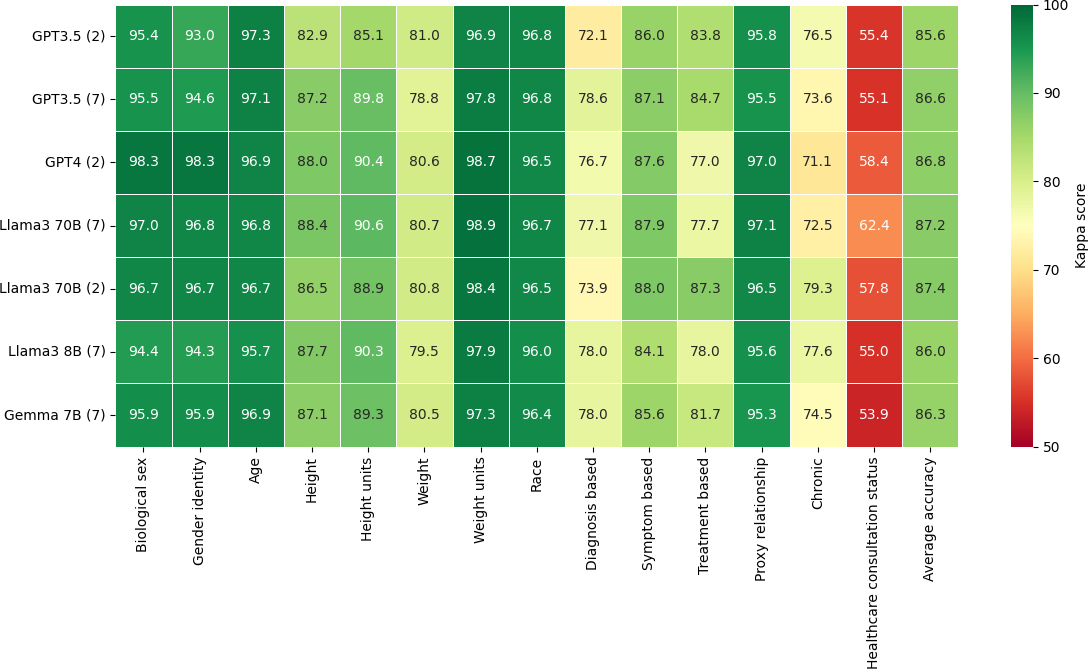
Detailed performance comparison of large language models in labeling AskDocs posts across various categories.

**Figure 3.**
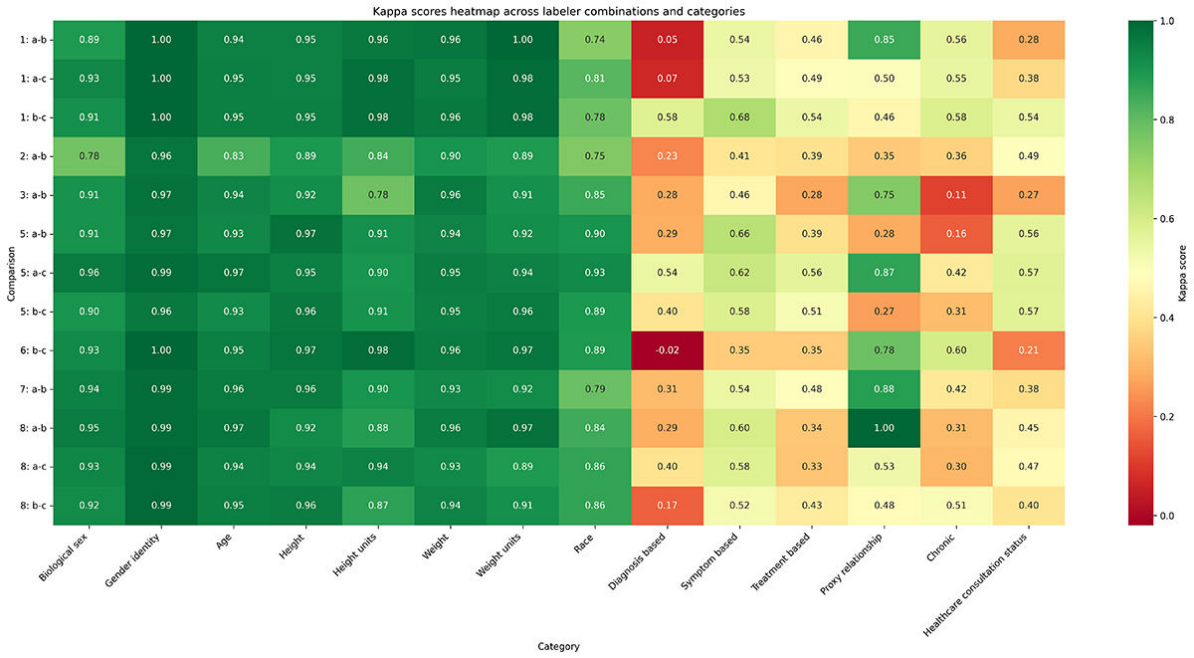
Cohen κ matrix displaying the agreement between different pairs of human annotators across the different categories of extraction. A higher κ score indicates a stronger agreement.

Human labeling was essential to this study, as it produced the gold standard dataset needed to evaluate LLM performance against human-level comprehension. The Cohen κ scores derived from the human annotators shed light on an inherent aspect of human-mediated data labeling: diversity in human judgment. While high levels of agreement in some categories (left side of Figure 2) validate the clarity of our guidelines, the variability in others (right side of Figure 2) reflects the natural divergence in human interpretation. This phenomenon serves as a crucial reminder that disagreements between the gold standard dataset and LLMs’ generation of labels do not inherently signify an error on the LLM’s part; rather, they may simply reflect the educated guesses that humans often make in the face of ambiguity. Crucially, the variability in human annotations, as visualized in Figure 3, is the reason multiple human annotators are necessary. The gold standard is created as an aggregate of the human annotations.

### High Disagreement Fields

As shown in Figure 3, the target questions exhibited varying levels of difficulty even for human annotators. The topics are categorized as Treatment-Based, Diagnosis-Based, and Symptom-Based. These fields aim to determine the reason for an individual’s post. Treatment-Based questions pertain to discussions about treatments, Diagnosis-Based questions relate to diagnoses, and Symptom-Based questions focus on symptoms. However, these categories can sometimes be ambiguous, as demonstrated by the following post:

*Dear doctors, I currently have a very minor case of poison ivy than that of my cases in the past; my girlfriend although believes it is contagious and refuses to make any contact with me (eg, Hold my hand). Is poison ivy contagious? I am a male, 18, and have had poison ivy for about 4 days now*.

In this instance, one annotator labeled the post as Diagnosis-Based, likely due to the mention of a poison ivy case, while the other 2 annotators classified it as Symptom-based, focusing on the symptoms of poison ivy.

Other challenging questions include those related to Chronic Conditions, Proxy Relationships, and Healthcare Consultation Status. Chronic Conditions questions inquire whether the issue is ongoing, Proxy Relationship questions address the poster’s relationship to the person with the issue, and Healthcare Consultation Status questions indicate Preconsultation, In Consultation, Postconsultation, depending on whether the individual has not yet seen a doctor, is currently seeing one, or has already done so. The following three items are examples of posts that led to disagreements among annotators:

1. “F23 test results came back and suggest a possible cyst. Should I pursue treatment? Here are the results. I originally went to the doc for lower back and side pain” (Consultation Status)
2. “Are these bug bites, and if so, should I be concerned? Here are some pictures of the bumps in question. The pictures are two weeks old, and I still have them.” (Chronic Condition)
3. “Do children need antibiotics for a UTI? Writing about a 7F. Has been needing to urinate per every few minutes. Luckily no pain when urinating and no blood either. Can this go away with cranberry juice and lots of water or are antibiotics needed?” (Proxy Relationship)

As an indicator of the LLM’s effectiveness, we use accuracy against the gold standard. Given the low agreement among human annotators on these topics, similar disagreements from LLMs reflect the inherent ambiguity within the dataset.

### LLM Labeling Process

To extract the structured data from the unstructured AskDocs subreddit, prompt engineering was used with the LLMs evaluated in this study. Prompt engineering is a critical process in the application of LLMs. It involves the careful design of prompts or instructions that guide the model in understanding and performing the desired task. The significance of prompt engineering lies in its ability to leverage the model’s inherent capabilities by translating the task at hand into a format that the model can comprehend and execute effectively, increasing the probability of receiving a correct response in the desired format. In this study, the techniques used in engineering prompts for this task were JavaScript Object Notation (JSON) fields formatting and few-shot prompting.

### JSON Fields Formatting

To comprehensively capture information shared by AskDocs subreddit users, a set of fields was defined within a JSON structure, a common format for representing data in a clear and accessible manner. Each field was designed to hold specific types of information, with permissible values outlined to ensure consistency and accuracy in the extracted data. The structured nature of JSON facilitated the straightforward combination of these fields into a cohesive dataset, where each post was transformed into a structured object. The same fields and values used by the human annotators were applied to the LLM outputs (Table 2).

### Few-Shot Prompting

Few-shot prompting with LLMs is an approach designed to enhance the models’ ability to perform specific tasks. Few-shot prompting involves creating a prompt that includes several examples of the task at hand (often in a Q/A or Input/Output pair format), followed by the new task to be completed. This technique effectively “primes” the model by providing it with a few examples of how to complete a specific task, thereby improving its ability to understand and execute similar tasks with new data. LLMs can perform few-shot prompting without fine-tuning, and Brown [20] showed that LLMs can perform numerous natural language processing tasks when provided a few examples in its prompt.

For instance, if the task involves extracting demographic information from unstructured health forum posts, a few-shot prompt might include examples as follows:

Example 1

Input: *I’m a 34-year-old male experiencing frequent headaches*.

Output: Age: 34, Gender: Male, Concern: frequent headaches.

Example 2

Input: *Female, 29, noticing a rash that appeared last week*.

Output: Age: 29, Gender: Female, Concern: rash appeared last week.

After presenting a few such examples, the model is then given a new, unseen piece of text and asked to perform the same task. This method capitalizes on the LLM’s ability to discern patterns and apply the learned extraction process to new data, enabling more accurate identification and categorization of information. Few-shot prompting thus represents a powerful tool in the prompt engineering tool kit, significantly enhancing the LLM’s use for data extraction [21]. This technique is crucial for several reasons:

- Flexibility: Few-shot prompting allows models to adapt quickly to new tasks without extensive model fine-tuning. Traditionally, adapting a model to perform a new or specific task necessitates a substantial investment in data labeling and computational resources for training, often making the process cost-prohibitive and time-consuming. Few-shot prompting, however, leverages the preexisting knowledge and versatility of LLMs, enabling them to understand and execute tasks with just a handful of examples.
- Consistency: Few-shot prompting helps in standardizing the output format, improving the odds that the LLM generates data in the defined JSON structure.
- Accuracy in information extraction: Previous work has shown that few-shot prompting of LLMs has the potential to drastically increase accuracy across a multitude of tasks of varying complexity.
- Proper JSON field creation: Although recent advancements allow enforcing JSON formatting through both OpenAI APIs and locally hosted models, these methods do not guarantee the generation of JSON objects with the correct fields. Few-shot prompting addresses this limitation by explicitly illustrating how each field should be populated, encouraging the model to produce objects with the appropriate field “keys.”

The prompt structure used in this study included (1) a brief introduction to the task, clarifying the goal of converting unstructured text into structured JSON format; (2) detailed instructions on how to approach the analysis, specifying the information that needs to be extracted and how it should be categorized into the JSON fields; and (3) examples to illustrate the labeling process, serving as templates for the LLM to follow. These examples demonstrate how to fill out each JSON field based on the content of the posts, ensuring clarity and precision in the output.

In this study, the researchers experimented with both 2-shot and 7-shot prompting techniques. Using only 2 or even 7 examples may seem limited; however, this is a defining feature of few-shot prompting. The way the prompt is crafted can significantly influence the results. Zhao et al [22] demonstrated that while increasing the number of examples can improve the accuracy of results, the gains tend to diminish as more examples are added. In addition, LLMs are susceptible to majority label bias, where the output is biased toward labels that are more frequent in the prompt. To mitigate this bias, examples were carefully selected that included a diverse range of labels, minimizing repetition wherever possible. All examples were taken from the training set and chosen prior to evaluation to avoid any overlap with test data. Labels were selected based on practicality, prioritizing diversity of content while keeping the full prompt within the model’s size limit.

By adopting few-shot prompting, the aim was to leverage the LLMs’ capabilities for consistent and accurate information extraction without expensive fine-tuning of the full LLM and to show the accuracy and the ability of these models to generalize for data extraction given just a few examples. In many respects, the LLMs were given the same amount of training examples as were the human annotators. The prompt used in our 7-shot prompting can be found in Multimedia Appendix 2.

### LLMs Evaluated

Four main types of models were used in this study: open-source models using Llama 3 from Meta [23] and Gemma from Google [24], along with proprietary models using GPT-3.5 [25] and GPT-4 [26] from OpenAI. The advantage of open-source models is that they are free to use, and the user has greater control of how the data are used and stored. Proprietary models, on the other hand, are perceived to be more accurate.

Llama 3 comes in 2 sizes: 8 billion parameters (7B) and 70 billion parameters (70B). Gemma is a 7 billion parameter (7B) model. Models with more parameters are usually more accurate, but they also require vastly more computing and storage resources to use. In comparison, GPT-3.5 has 175 billion parameters, and as there is no official disclosure on its size, it can be assumed to be much larger. The analysis used few-shot prompts containing 2 examples for all models except for GPT-3.5, which was run using both 2 examples and 7 examples. Two examples were used for GPT-4 due to the high cost of adding more examples. The Llama 3 and Gemma models were run using various sizes of context for comparison.

### Ethical Considerations

There are several ethical considerations when using LLMs to analyze health-related data. While Reddit posts are publicly available, users may not expect them to be used for medical research, and many posts may contain sensitive personal health-related information. In this study, no usernames, post IDs, or other identifying metadata were included in the analysis, with all data deidentified to reduce privacy risk. In addition, the research adhered to Reddit’s API use policy. As LLMs become increasingly deployed for health-related research, it is essential to maintain strict adherence to privacy regulations such as the Health Insurance Portability and Accountability Act (HIPAA), obtain institutional review board approval when applicable, and consider on-premises model deployment (running LLMs locally rather than through a third party) to avoid the risk of leaking of sensitive data. IRB approval for this research was proposed, approved and classified as exempt level, category 4: Secondary research for which consent is not required.

## Results

### LLM Labeling Compared With Human Annotators

After running each model, the accuracy score (percentage agreement) was computed for the LLM results versus the gold standard (Figure 2). In terms of agreement with the gold standard, the top-performing models were the largest, with GPT-4 (2-shot) and Llama 3 70B (7-shot) having the highest overall accuracy. It appears that in general, GPT-4 had the highest agreement with the gold standard in the simpler fields, such as Biological Sex, Gender Identity, and Age, while Llama 3 70B performed marginally better in more subjective fields, with slightly higher percentage agreement scores in Diagnosis Based, Symptom Based, Treatment Based, and Chronic. However, this performance difference may be attributed to the larger number of few-shot examples used in the Llama 3.

One key finding was the agreement of LLMs in more subjective areas, such as determining whether a condition was chronic or assessing the health care consultation status. Like human annotators, LLMs encountered challenges in these subjective categories, reflecting the inherent complexity and nuanced understanding required to make these determinations.

**Figure 4.**
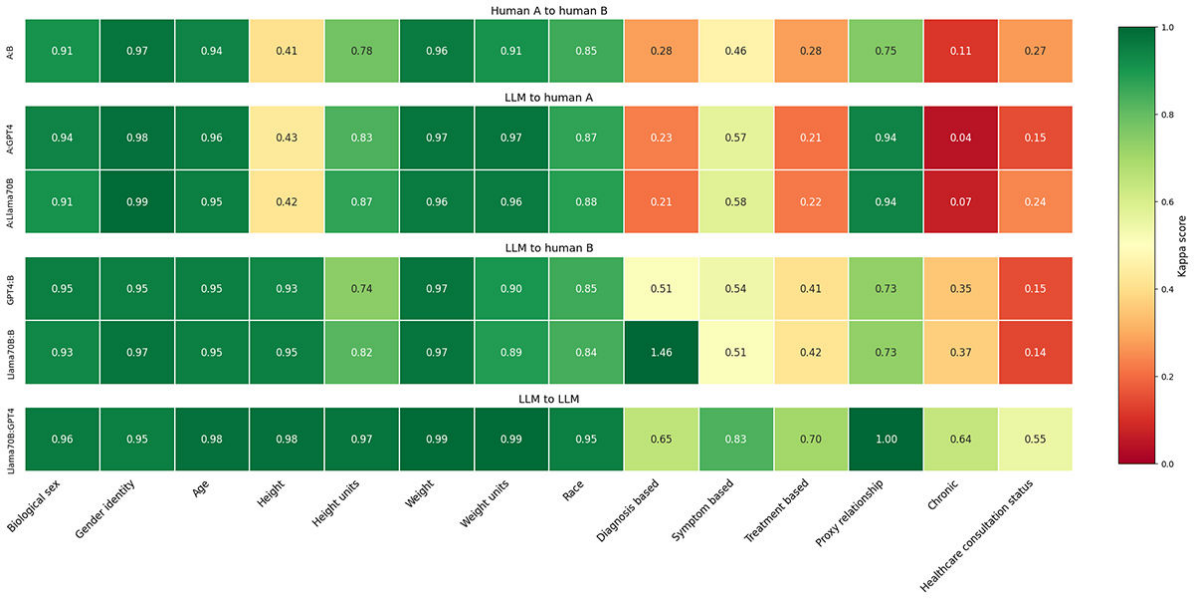
Cohen κ scores: GPT-3.5 versus gold standard dataset and between 2 human annotators. LLM: large language model.

Moreover, a side-by-side comparison of Cohen κ scores between the top 2 performing LLMs (GPT-4 and Llama 3 70B) and a randomly selected pair of human annotators revealed similarities in the pattern of disagreements. These results, shown in Figure 4, show that the differences between either LLM or annotator A resemble the difference between annotators A and B. This observation suggests that the discrepancies between LLM outputs and the gold standard dataset may mirror the natural variance found in human labeling efforts. This highlights the capabilities of LLMs to approximate human-like understanding and judgment in complex categorization tasks.

**Figure 5.**
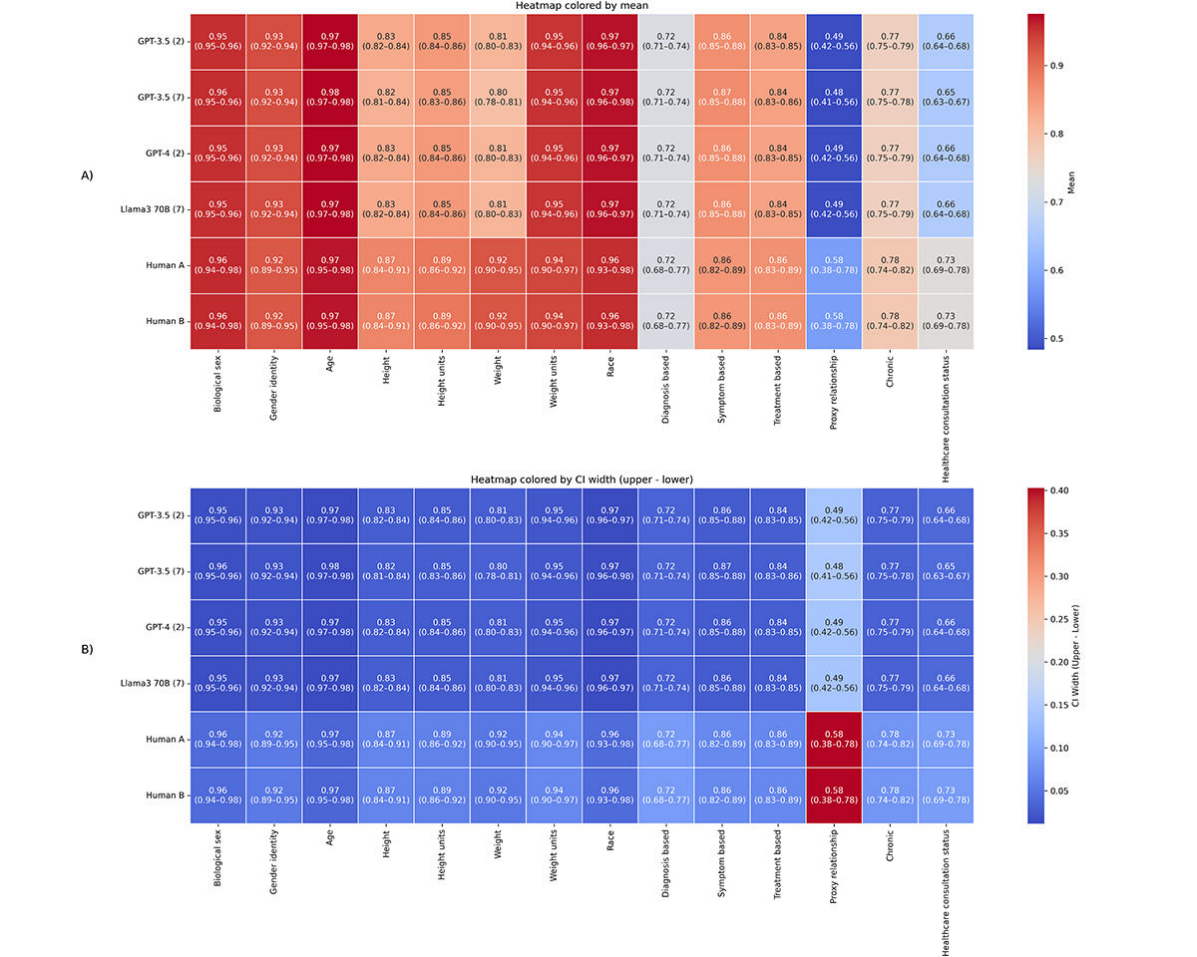
Agreement with gold standard 95% confidence interval heatmaps colored by mean value (A) and confidence interval range (B).

A more detailed understanding of the differences between human and LLM data extraction was gained by examining the confidence intervals for agreement with the gold standard across each data field. Figure 5 shows heatmaps of the confidence intervals. The top heatmap (A) is colored by the mean value of the confidence interval, while the lower heatmap (B) is colored by the confidence interval ranges. As seen previously, more subjective data fields tend to have lower mean values and wider confidence intervals. Notably, LLMs showed greater consistency than the 2 human annotators. The LLM confidence interval ranges are, in general, tighter than those of the human annotators. Of note is the Proxy Relationship data field. As mentioned previously, this is a particularly difficult data field to extract from the information in the posts. All the LLMs seem to struggle in the same way. Human annotators have a significantly larger confidence interval range. This may be due to human annotators making more judgment calls based on other information in the post.

### Demographic Insights and Health Discourse Trends

With the methodology established, it was applied to approximately 30,000 randomly sampled Reddit posts using GPT-3.5. GPT-3.5 was used in this analysis because it offered a balance of speed, cost-efficiency, and accuracy for large-scale processing at a lower cost and time compared with GPT-4. This large-scale analysis demonstrates the unique insights that can be obtained from the health discourse on the AskDocs subreddit related to age distribution, the nature of inquiries, proxy relationship posts, and health topics discussed. This section serves to demonstrate the strengths of using LLMs to extract data that would be otherwise impractical using human extraction. It focuses on the capabilities of the data extraction method. Future research may involve a more comprehensive analysis, including treatment of missing or incomplete data.

### Age Distribution

**Figure 6.**
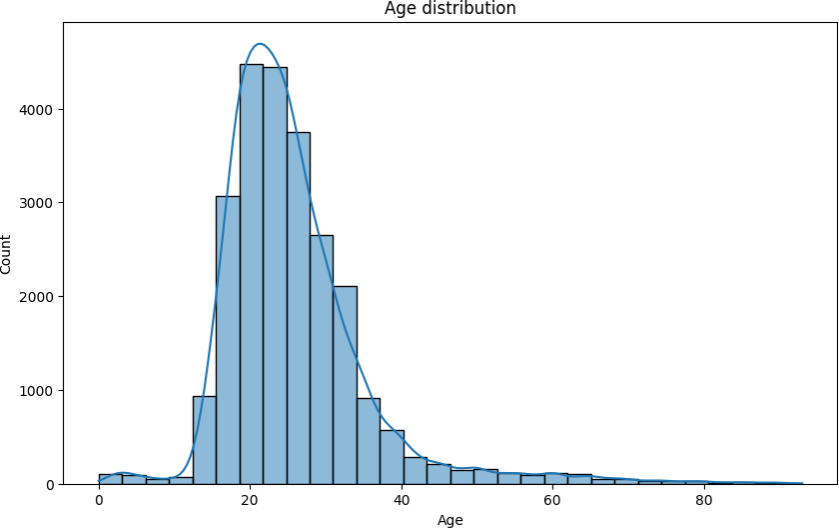
Age distribution of AskDocs users.

Figure 6 illustrates the age distribution of users partaking in the AskDocs subreddit. The visible skew toward a younger demographic may reflect a generational trend in using online platforms for health-related guidance. This observation aligns with broader usage patterns on Reddit, where 44% of users are aged between 18 and 29 years, and 31% are aged between 30 and 49 years [27], suggesting that the demographic trends observed in AskDocs may indeed be representative of the general Reddit user base.

### Nature of Inquiry

When the posts are segmented by the nature of the inquiry: Diagnosis-Based, Treatment-Based, or Symptom-Based, the results show that 85% are diagnosis-based posts, 66% contain treatment-based information, and 93% contain symptom-based information. Note that a single post may contain any or all 3 of the categories. The predominance of Symptom-Based queries suggests that users are often at an initial stage of seeking health information, which may involve checking symptoms, sometimes anonymously, before seeking formal medical consultation.

### Proxy Relationship Posts

The vast majority (95%) of inquiries are made by individuals concerning their own health, highlighting the subreddit’s predominant role in facilitating personal health inquiries. When queries are made on behalf of others, they are predominantly for significant others or children (Table 4).

**Table 4.** Proxy relationship posts as a percentage of total posts.

Proxy relationship	Percentage of all posts
OP’s^a^ significant other	1.765
OP’s child	1.459
OP’s friend	0.257
OP’s sibling	0.095
Other	0.063
OP’s parent	0.025
OP’s relative	0.007

a OP’s: original poster’s.

### Health Topics

**Figure 7.**
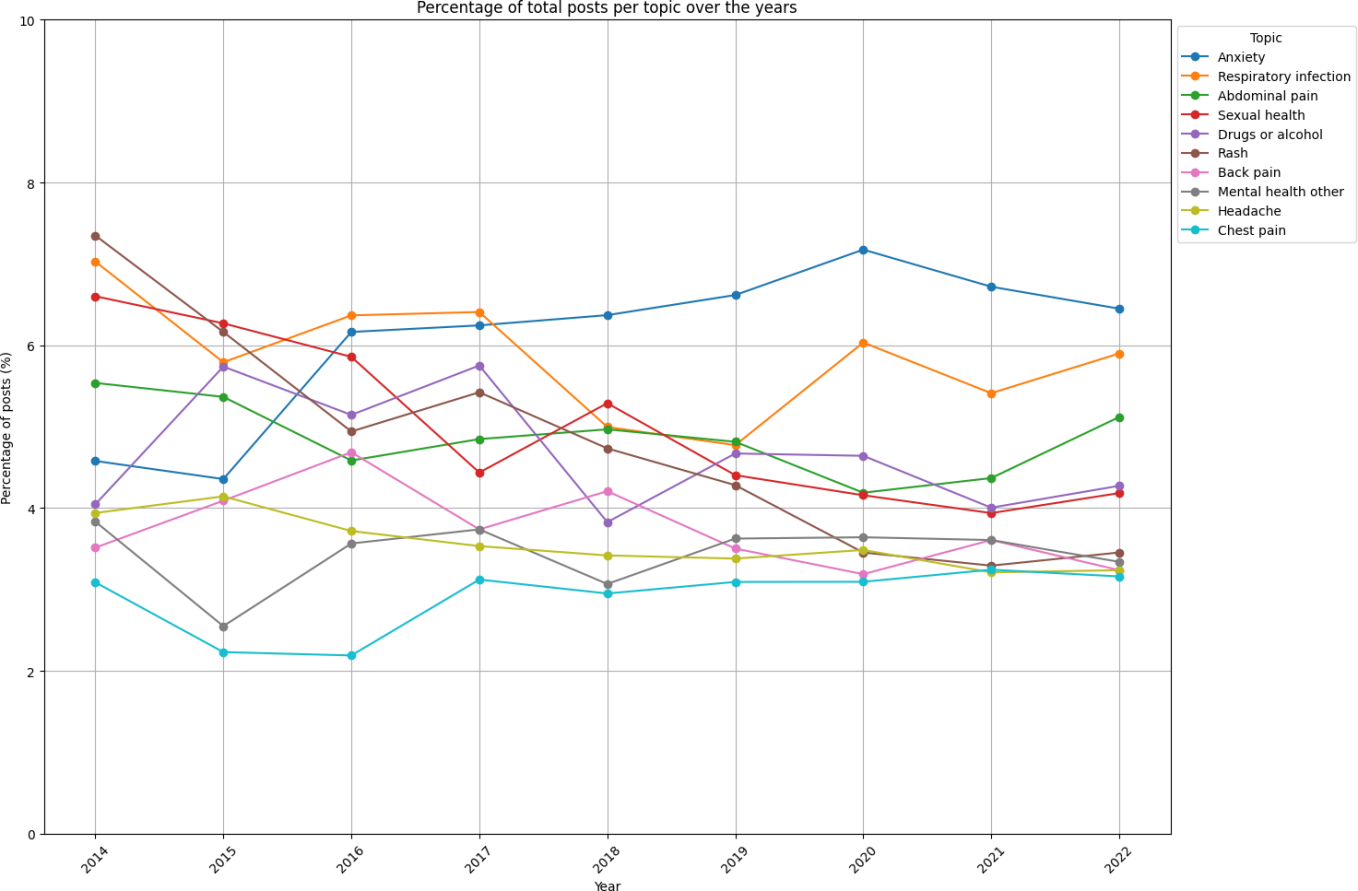
Most common health topics discussed on AskDocs as a percentage of all posts over time.

Finally, Figure 7 details what percentage of posts pertained to each of the top 10 most frequently discussed health topics. Notably, “Anxiety” and “Respiratory Infections” both saw marked increases in discussion volume from 2019 to 2020, likely influenced by the COVID-19 pandemic, reflecting public health trends and possibly exacerbated public anxieties [28].

## Discussion

### Principal Findings

This study aimed to assess the application of LLMs in systematically converting the rich, unstructured textual data from the Reddit r/AskDocs subreddit into a structured dataset, a method that more closely mirrors human cognitive processes than conventional data extraction techniques. This comparative analysis shed light on the efficacy of labeling via LLMs relative to that of human annotators in the nuanced domain of a web-based health forum such as Reddit. The insights garnered point toward both the strengths and limitations of current AI technologies in domain-specific content understanding, paving the way for further research and development in the field of digital health communication.

To further demonstrate the potential of the LLM-labeled data, a cursory analysis was conducted that revealed patterns and trends within the AskDocs subreddit community. Insights such as these have the potential to guide public health research, tailor medical advice services, and support targeted health information dissemination, although further validation across more diverse datasets and additional forums is necessary for broader applicability and verification of these results.

The findings shown in Figure 4 indicate that Llama 3 70B with a 7 few-shot prompt and GPT-4 with a 2 few-shot prompt had the highest agreement with benchmark human-annotated data among the models run. Due to financial constraints, GPT-4 with a 7 few-shot prompt was not run. These results reflect the advanced capabilities of these models to understand and process complex health-related information. Few-shot examples may enhance the performance of LLMs by improving their ability to recognize specific patterns, as they help the model interpret tasks more accurately [20,29].

It is important to note that Llama 3 70B is an open-source model, which allows it to be downloaded and run locally without incurring additional costs. This feature becomes particularly significant when considering information privacy or scenarios where data sensitivity prohibits the use of web-based servers. Furthermore, the performance of the open-source Llama 3 70B is comparable with its proprietary counterpart, thereby enabling the application of these techniques in research contexts where such resources might otherwise be unavailable.

The pattern of disagreement between LLMs versus the gold standard and human annotators versus the gold standard exhibited notable similarities. Fields where human annotators had low agreement with the gold standard, as depicted in Figure 2, also posed challenges for LLMs. This suggests that the complexity of these questions may explain reduced LLM agreement, reflecting the same areas of human disagreement in the annotated dataset. For instance, fields with high disagreement among human annotators also showed low agreement between human annotators and LLMs, as shown by decreased accuracy. Conversely, fields where humans achieved high Cohen κ scores, such as Biological Sex, also demonstrated high accuracy from LLMs.

This notion is further supported by the results illustrated in Figure 5. The comparison of Cohen κ scores between our LLMs with the highest percentage agreement with the gold standard and a randomly selected pair of human annotators revealed that the pattern of disagreement between LLMs and human annotators mirrored the disagreement among the annotators themselves. This suggests that the consistency of LLMs is comparable with that of human annotators. It further indicates that LLMs can approximate human judgment, although perfect coding remains unlikely in subjective categories for both LLMs and humans. Therefore, when human annotators disagree with similar consistency as an LLM does with them, it may be reasonable to consider the LLM annotations with the same weight as those made by human annotators.

The ability of LLMs to extract health data from large-scale, unstructured sources such as Reddit posts has implications for clinicians and public health researchers. For example, health care providers may use the AskDocs subreddit to identify concerns related to emerging health issues, explore health-related misconceptions, and monitor the side effects of medications. From a public health perspective, understanding the health-related issues and their determinants is important for informing the development of interventions such as health communication campaigns, educational programs, policy initiatives, and environmental changes. In addition, LLMs could provide real-time health sentiment analysis, which public health organizations could leverage to improve interventions in response to changing conversations and attitudes in web-based communities.

### Operational and Economic Analysis

The utilization of LLMs for data extraction, especially within the domain of health care and online forums such as the AskDocs subreddit, offers advantages over traditional manual methods in terms of efficiency, consistency, scalability, and privacy.

Unlike human annotators, LLMs can analyze and extract data from thousands of documents in a fraction of the time. Rapid processing is valuable for time-sensitive tasks such as monitoring health forums for emergent public health concerns or extracting patient information from clinical notes in real time. Unlike humans, whose performance may fluctuate due to fatigue or subjective interpretation, LLMs maintain a high degree of reliability and consistency in data extraction tasks.

Scalability is another benefit, as LLMs can be parallelized and deployed across multiple servers, allowing for simultaneous processing of data from various sources. In addition, LLMs can be configured to extract relevant information while preserving user anonymity and discarding sensitive personal data, thus supporting compliance with regulations such as HIPAA [30].

The costs and time required based on the current OpenAI API’s tier 5 rate limit and token pricing [31] are shown in Table 5. From an economic and operational standpoint, LLMs are very efficient. For example, labeling 30,000 posts manually at 1 post per minute would take 500 hours and cost approximately US $7500 at a rate of US $0.25 per post. In contrast, GPT-3.5 Turbo can perform the same task at a cost of only $285 and in just more than 4 hours, processing 117 posts per minute. The substantial cost and time efficiencies of GPT-3.5 Turbo compared with both human annotator and the more expensive GPT-4 model are notable. The ability to process large datasets quickly and economically with GPT-3.5 demonstrates its advantage for users needing high throughput and cost-effectiveness, whereas GPT-4 offers advanced capabilities at a higher expense. As the LLM landscape progresses, the costs associated with these technologies are expected to fluctuate, potentially making high-capability models such as GPT-4 more accessible.

**Table 5.** Cost and time comparison in US dollars for labeling 30,000 posts using different methods.

Method	Cost per post	Total cost	Posts per minute	Total time (hours)
Human	$0.25	$7500	1	500
GPT-3.5 Turbo	$0.0095	$285	117	4.27
GPT-4	$0.54	$16,200	18	28.33
LLama	Free	Free	19	26.31

### Limitations and Future Research

Despite the demonstrated strengths of using LLMs in health-related information extraction, this research has limitations that pave the way for future research opportunities. LLM research is a rapidly advancing field, with new models and techniques regularly emerging. The methodologies used in this study could be refined by integrating state-of-the-art models and approaches that have been developed since the time of our research. Ongoing research should integrate the latest advancements to enhance the accuracy, efficiency, and reliability of data extraction processes.

Furthermore, the study faced financial limitations that restricted our ability to fully use GPT-4 with n-shot samples. Although data trends indicated that the models performed better with an increased number of n-shot samples, we were unable to conduct extensive experimentation with GPT-4 to its fullest potential. Consequently, the results might have been enhanced if we had the resources to conduct more extensive testing with additional n-shot samples.

A significant area for future research lies in applying these methodologies to analyze HIPAA-protected data. Currently, accessing such data involves complex legal processes to ensure privacy and compliance. By processing these data through an LLM, it may be possible to effectively extract the information without direct human access to protected information, thereby facilitating analysis that was previously hindered by legal and ethical constraints. Research exploring the extent to which LLMs can maintain data anonymity while still providing valuable insights would be highly beneficial. In addition, research should explore technical solutions such as on-premises deployment (running LLMs on local servers) to minimize exposing sensitive data and follow ethical guidelines for using AI in health care.

The introduction of new models with more extensive context lengths (allowing for longer prompts) provides an opportunity to include more examples in few-shot prompting, which may improve the model’s understanding and execution of the data extraction task. Investigating whether the incorporation of more examples enhances the model’s performance would provide valuable insights into the few-shot learning capabilities of LLMs. This research could involve experimental comparisons between models with varying context lengths to determine the optimal number of examples for accurate data extraction.

While this study targets the medical domain, particularly the AskDocs subreddit, the methodologies used can and should be validated in a broader range of domains. Future research should extend beyond health forums to encompass a wide array of fields, creating large-scale datasets and using LLMs for data extraction in each context. Comparing the performance of LLMs with expert human annotators and established automated methods across these varied domains is essential. This expanded benchmarking will not only solidify our understanding of LLMs’ practical limitations but also verify their reliability and adaptability to diverse applications. Such cross-domain validation will underscore the versatility of LLMs and inform their refinement for specialized tasks.

Finally, the user base of Reddit is generally younger and more technologically literate, which may bias these data toward population segments that may not reflect broader public demographics. As such, any policy-related decisions should be made in the context of the demographics of this study.

### Conclusions

While this study has laid the groundwork for understanding the strengths and limitations of the use of LLMs in extracting structured data from unstructured text, there remains significant potential for further exploration. In this study, LLMs’ performance was comparable with human annotators in extracting demographic and health-related information from unstructured posts on the AskDocs subreddit. LLMs offer many advantages, including scalability, consistency, and cost-efficiency, while processing tens of thousands of posts in a fraction of the time and cost required for manual annotation. However, both LLMs and human annotators struggled with subjective labeling fields, underscoring the complexity and ambiguity of some health-related posts. Further in-depth study surrounding the characterization of misclassifications and various model-specific weaknesses is part of ongoing research. Despite these challenges, accuracy in objective feature extraction and consistency in LLM and human disagreements for subjective features suggest that LLMs can be viable, scalable tools in digital health research. Continuous advancements in LLM technology, combined with rigorous research into their applications and implications, will enhance understanding and contribute to the evolution of digital health care research.

## Supplementary material

10.2196/74094 Multimedia Appendix 1 Labeling guidelines.

10.2196/74094 Multimedia Appendix 2 Example prompt for a large language model.

## Abbreviations

AI: artificial intelligence
API: application programming interface
HIPAA: Health Insurance Portability and Accountability Act
JSON: JavaScript Object Notation
LLM: large language model
