# Supplementary material for: Assessing Large Language Models in Building a Structured Dataset From AskDocs Subreddit Data: Methodological Study
Source: J Med Internet Res. 2025 Oct 22;27:e74094. doi: 10.2196/74094 (PMC12543290; doi:10.2196/74094)
Supplement: Multimedia Appendix 1 [file jmir-v27-e74094-s001.docx]

**Multimedia Appendix 1 – Labeling Guidelines**

**Introduction:**
Thank you for your participation in this exciting research! I’ve created this document to better help you understand your task in labeling these documents and moving this research forward.

**A unique Google Sheet has been shared with you** containing 400 rows, each row containing the id, title, and body of a post from r/askdocs, a popular online forum where users ask medical questions for medical professionals to answer at their leisure.

**Your task is to read each post’s title and selftext (body) and fill out information for each of the different fields on the sheet.** I anticipate that it will take roughly 10 hours to label all 400 posts. When you’ve completed your assigned labeling, please contact me and let me know.

Our goal is to complete this stage of the research quickly, so let’s make **Wednesday, June 28th EOD our deadline.** If you’ll be unable to reach this deadline, please contact me.

Let's dive into the specifics. It’s crucial to understand the different fields you will be labeling in this process. Each field corresponds to a particular aspect of the information contained in the posts. Below, in the **Field Descriptions** section, you will find detailed explanations for each field. Once you are acquainted with these fields, we will guide you through the **mechanics of labeling on the Google Sheet**, which has been optimized to streamline your workflow. We will also offer some **Recommendations and Reminders** for best practices during the labeling process. Finally, I have created a few **Examples** that will help prime you for the work you’re setting out on.

**Field Descriptions:**

- **Biological Sex (M, F, Unknown, NA):** The biological sex of the subject.
- **Gender identity (M, F, Other, NA):** The gender identity of the subject.
- **Age (Number, Unknown):** The age of the subject (in years).
- **Height (e.g., 6'0", 170, Unknown):** The height of the subject.
- **Height Units (feet/in, cm, m, NA.):** The units in which the subject’s height is given. (Can be assumed by context)
- **Weight (Number, Unknown):** The weight of the subject.
- **Weight Units (lbs, kg, NA):** The units in which the subject’s weight is given. (Can be assumed by context)
- **Race (White, Asian, Black, Hispanic, Other, Unknown):** The racial background of the subject.
- **Diagnosis Based (True, False, NA):** Indicates if the post is focused on understanding or discussing a specific diagnosis.
- **Symptom Based (True, False, NA):** Indicates if the post is centered on symptoms experienced by the subject without a known cause.
- **Treatment Based (True, False, NA):** Indicates if the post is about a particular medication or treatment.
- **Proxy Relationship (NA, OP’S Significant Other, OP’s Friend, OP’s Child, etc., Other):** If this post is on behalf of another person, this field should include the relationship that the subject (the one for whom the question is being asked) has with the Original Poster (the author of the post). If the post is not on behalf of another person, it should be marked NA.
- **Chronic (True, False, NA):** Indicates if the medical condition mentioned in the post is chronic, meaning it is persistent or long-lasting.
- **Healthcare Consultation Status (Pre-Consultation, In Consultation, Post-Consultation, NA):** Indicates the subject's stage in the healthcare consultation process at the time the post was made.
- **Topic (Dropdown menu)**: This category indicates the primary reason or focus of the post, based on a dropdown list of common medical topics. Labelers should select one or more topics that directly relate to the user's main concern or query. It is important to focus on the central issue the user is posting about, rather than unrelated elements of their medical history. For instance, in a post mentioning a past cancer diagnosis, current medication, and a recent car accident, but primarily seeking advice on the accident, the label should be "Accident". Multiple topics can be selected if the post addresses more than one primary concern.

**Options for the “Topic” Category:**

- **Anxiety:** Posts discussing anxiety disorders or feelings of excessive fear and worry.
- **Back pain:** Posts about pain in the back, typically related to muscles or bones, excluding symptoms of other conditions.
- **Depression:** Posts discussing depressive disorders or symptoms like prolonged sadness or loss of interest.
- **Drugs or Alcohol:** Posts discussing recreational drug or alcohol use, regardless of legality or evidence of abuse.
- **Earache:** Posts about pain in the ear or inner ear infections, excluding issues with the external ear.
- **Headache:** Posts primarily focused on pain in the head, excluding headaches mentioned as secondary symptoms.
- **Hypertension:** Posts discussing high blood pressure and its management or treatment.
- **Irritable Bowel Syndrome (IBS):** Posts about IBS, including symptoms like abdominal pain, bloating, and changes in bowel habits.
- **Rash:** Posts primarily about skin rashes, regardless of the known or unknown cause.
- **Sexual Health:** Posts regarding issues related to pregnancy, fertility, sexual performance, or sexually transmitted diseases.
- **Respiratory Infection:** Posts about infections affecting the respiratory system, such as colds, the flu, or throat infections.
- **Urinary Tract Infection:** Posts discussing symptoms or diagnosis of infections in the urinary tract.
- **Vomiting or Diarrhea:** Posts about acute gastrointestinal infections characterized by vomiting or diarrhea.
- **Suicidality:** Posts discussing thoughts, attempts, or acts of self-harm or suicide.
- **Mental Health Other:** Posts about mental health issues not covered under anxiety or depression, such as OCD, bipolar disorder, or schizophrenia.
- **Accident:** Posts regarding physical injuries due to accidents, not resulting in death.
- **Chest pain:** Posts discussing pain in the chest area, which may be related to heart, lungs, or other structures.
- **Shortness of breath:** Posts about difficulty breathing or feeling out of breath.
- **Abdominal pain:** Posts focusing on pain in the stomach area, excluding specific conditions like IBS.
- **Other:** Posts that do not fit into the above categories or discuss various miscellaneous topics.

**Mechanics of Labeling on the Google Sheet**:
These functionalities have been implemented to streamline the labeling process, making it easier to manage data and minimize manual input errors. Note that some of these automated processes take a half second to trigger.

- **Auto-Filling Gender Identity:** When you input the biological sex in Column D (Male, Female, Unknown, or NA), the script will automatically fill the corresponding Gender Identity in Column E. For example, if you select “Male” as the Biological Sex, “Male” will be automatically filled in the Gender Identity column. This saves time by eliminating an extra step for the cases in which subjects are cisgendered.
- **Multi-Selection in “Topics”:** The Google Sheet is designed to allow you to select multiple options from the dropdown in Column R. When you select an item, it gets added to the cell. If you want to add more items, simply click the drop down again and select another option. The additional topic will then be appended to the cell separated by a comma. If you’d like to restart or remove an item from the list, simply double click the cell and manually edit the text.
- **Hiding Completed Rows:** There is a checkbox in Column S. When you are finished with a row and want to hide it for a clearer view of the remaining data, you can check this box, and the row will be hidden from view.
- **Unhiding All Rows:** In Row 1, Column U, there is a master checkbox. If you want to make all hidden rows reappear at once, you can check this box. Note that after clicking it, the checkbox will automatically uncheck itself, but the rows will remain visible.
- **Default Values:** The default values of the rows are such that if a user doesn’t mention something, the value can usually just remain as is. For example, if a user doesn’t mention their own demographic information, you can proceed without touching the demographic section, since the values are all Unknown/NA by default.

**Recommendations and Reminders:**

- **Utilize Dual Monitors:** For enhanced productivity and reduced chances of error, it is highly recommended to use two monitors – one for reading the posts and the other for keying in the data. This setup minimizes the need for scrolling and provides a more ergonomic workflow.
- **Take Breaks:** Labeling can be a monotonous and strenuous task. As fatigue sets in, the accuracy of labeling may decrease. It is essential to take regular breaks to ensure the quality of your work stays high.
- **Consistency in Labeling:** Pay attention to consistency in labeling, especially when handling similar posts. Consistent labeling helps in maintaining the integrity of the dataset.
- **Review Guidelines:** Familiarize yourself with the labeling guidelines and keep them handy. It's easy to overlook or forget details, and having the guidelines nearby can be a helpful reference.
- **Using Titles and Selftext:** In many cases, a user has included vital information in the title of a post, and not in the selftext (body). Don’t forget to check the title!

**Examples:**Below are a few examples I have collected and the labels that would be given for each one. I’ve included brief explanations on why each label was chosen to help you better understand the process.

Note that because the data is pulled straight from reddit, there are some vestigial formatting and character codes (&amp;#x200B;) that do not transfer well to Google Sheets. These can be ignored.  **Example 1:**

id: bqv00p
title: Might be a blood clot?

selftext: Age 15 ,Male, 5'8, 215 lbs, is being Mexican considered a race? have had this pain for the past day or so. have no current medications besides pills my grandpa gives me that supposedly help manage my above average cholesterol. I've had asthma for my entire life. I use no recreational drugs and I don't smoke. &amp;#x200B; &amp;#x200B; Today I've noticed that my thigh has been hurting. The feeling is very hard to describe. The closest I've come to describing accurately is a "gurgle" and tight feeling area in the back of my thigh (about 3 inches above the back of my knee) any help would be appreciated. It's slowly getting more painful. I am currently in the process of losing weight. &amp;#x200B; If this does sound like a blood clot I will go to the hospital immediately.

**Biological Sex: M** (The user mentions they are Male)

**Gender identity: M** (Automatically filled based on Biological Sex)

**Age: 15** (The user mentions they are 15 years old)

**Height: 5'8"** (The user mentions they are 5'8")

**Height Units: feet/in** (The height is given in feet and inches)

**Weight: 215** (The user mentions they weigh 215 lbs)

**Weight Units: lbs** (The weight is given in pounds)

**Race: Hispanic** (The user mentions being Mexican, which is considered Hispanic)

**Diagnosis Based: False** (The post is not focused on understanding or discussing a specific diagnosis)

**Symptom Based: True** (The post is centered on symptoms experienced by the subject, such as pain in the thigh)

**Treatment Based: False** (The post is not about a particular medication or treatment)

**Proxy Relationship: NA** (The post is written by the subject themselves, not on behalf of someone else)

**Chronic: False** (The post mentions pain for the past day, not persistent or long-lasting)

**Healthcare Consultation Status: Pre-Consultation** (The user is concerned about symptoms and mentions they will go to the hospital if it's serious, but hasn't consulted a healthcare professional yet)

**Topic: Other** (The post does not fit into the categories given, as it focuses on a pain in the thigh, which is not listed in the topic options)

**Example 2:**

id: cir0as
title: 6 day old newborn won't wake up
sefltext: My wife and I had our baby girl 6 days ago. She has since been losing weight (as is expected for newborns). We have her on a feeding schedule to eat every 2-4 hours. Tonight we have been having difficulty waking her up to feed. We tried once, but she wouldn't wake up, so we decided to let her go another hour. After that hour, we tried waking her again, and she won't wake up. Please keep in mind she is still breathing, and there are no other concerns other than her not waking up. We have tried stripping her down, changing her diaper (she did have a dirty diaper during this period, which we changed), turning on all the lights, and making noise, but still nothing. Should I be worried and take her to an ER?

**Biological Sex: F** (The post mentions that the baby is a girl.)

**Gender identity: F** (Automatically filled based on Biological Sex.)

**Age: 0** (The post mentions that the baby is 6 days old, so age is 0 years.)

**Height: Unknown** (The post does not mention the height of the baby.)

**Height Units: NA** (The height is not mentioned.)

**Weight: Unknown** (The post mentions the baby is losing weight but doesn't give a specific number.)

**Weight Units: NA** (The weight is not mentioned.)

**Race: Unknown** (The post does not mention the racial background of the baby.)

**Diagnosis Based: False** (The post is not focused on understanding or discussing a specific diagnosis.)

**Symptom Based: True** (The post is centered on a concern regarding the baby not waking up for feeding.)

**Treatment Based: False** (The post is not about a particular medication or treatment.)

**Proxy Relationship: OP’s Child** (The post is written by a parent on behalf of their newborn child.)

**Chronic: False** (The post mentions an issue occurring that night, not persistent or long-lasting.)

**Healthcare Consultation Status: Pre-Consultation** (The post is asking for advice on whether they should take the baby to an ER, indicating they haven't consulted a healthcare professional yet.)

**Topic: Other** (The post focuses on a concern about the newborn baby not waking up for feeding, which is not listed in the topic options.)

**Example 3:**id: jocfuo
title: Autoimmune maybe?

selftext: Age: 25 Female 160 lbs 5’4 [leg photos ](https://imgur.com/a/WpPczeg) I got these all over my legs, thighs, butt. They started after a respiratory infection. (I’ve gotten these tons growing up) they started small, grew into these raised clusters. When you push on them they didn’t turn white. Always stay dark red. When my calf and thigh touched, they burned. They finally went away after a couple weeks but then hyper pigmented my legs which still are slightly. Doctors thought maybe igA vasculitis. But my blood work came back fine with high white blood cells. They just sent me on my way. There’s clearly something wrong and now have spots everywhere hat are hyper pigmented. Also I now (it’s been slowly coming on but way worse after this, have ankle swelling that hurts, ans knee, and hand aching issues. Also: (I’ve always had weird skin issues, they say seborrheic dermatitis, my skin peels like Eczema on my hands constantly, I get skin infections easily and respiratory. Also get hives easily in warm areas if my body heat rises too quickly ) Doctors I’ve been to aren’t figuring it out, and I am seeing another one tomorrow. Any advice would be greatly appreciated!

**Biological Sex: F** (The user mentions they are Female)

**Gender identity: F** (Automatically filled based on Biological Sex)

**Age: 25** (The user mentions they are 25 years old)

**Height: 5'4"** (The user mentions they are 5'4")

**Height Units: feet/in** (The height is given in feet and inches)

**Weight: 160** (The user mentions they weigh 160 lbs)

**Weight Units: lbs** (The weight is given in pounds)

**Race: Unknown** (The post does not mention the user's racial background)

**Diagnosis Based: True** (The post mentions doctors' thoughts on potential diagnoses like IgA vasculitis)

**Symptom Based: True** (The post describes various symptoms, such as raised clusters on legs, burning sensation, hyperpigmentation, and swelling)

**Treatment Based: False** (The post is not focused on a particular medication or treatment but rather seeking advice)

**Proxy Relationship: NA** (The post is written by the subject themselves, not on behalf of someone else)

**Chronic: True** (The post mentions issues that have been persistent and have occurred multiple times)

**Healthcare Consultation Status: In Consultation** (The user mentions having been to doctors and is going to see another one)

**Topic: Rash, Other** (The post describes skin issues including raised clusters, burning, hyperpigmentation, which falls under Rash, and mentions other issues that do not fit under any other specific category)

Example 4:

id: f5sue4

title: 29F, various symptoms for over a year

selftext: delete

**Biological Sex: F** (The title mentions that the subject is a 29F, indicating Female.)

**Gender identity: F** (Automatically filled based on Biological Sex)

**Age: 29** (The title mentions that the subject is 29 years old.)

**Height: Unknown** (The post does not mention the subject's height.)

**Height Units: NA** (Not applicable as height is not mentioned.)

**Weight: Unknown** (The post does not mention the subject's weight.)

**Weight Units: NA** (Not applicable as weight is not mentioned.)

**Race: Unknown** (The post does not mention the subject's racial background.)

**Diagnosis Based: Unknown** (The post content is "delete", so it is unknown if the post was intended to discuss a specific diagnosis.)

**Symptom Based: True** (The title mentions "various symptoms", indicating the post is symptom-based.)

**Treatment Based: Unknown** (The post content is "delete", so it is unknown if the post was intended to discuss a particular medication or treatment.)

**Proxy Relationship: NA** (The post is likely written by the subject themselves as indicated by the title, not on behalf of someone else.)

**Chronic: True** (The title mentions "over a year", indicating that the symptoms are persistent or long-lasting.)

**Healthcare Consultation Status: Unknown** (The post content is "delete", so it is unknown if the subject has consulted a healthcare professional.)

**Topic: Other** (The post does not provide sufficient information to categorize it under any specific topic.)

**Example 5:**

id: 2kbjds

title: Swelling symptoms
selftext: I'm a 55 year old caucasion male. 6'3" 275lbs. I've been diagnosed with a shattered heel bone 1(occured on Saturday 10/18) with surgery scheduled in 2 weeks. The orthopedic surgeon wants to wait for the swelling to go down before surgery. My question has to do with the symptoms of the swelling. As the swelling has begun to subside the normal color changes have occurred. However, I've developed a red, irritated area on the top of my foot that burns when touched or comes in contact with my bandages. The burning can be described as feeling like the skin is on fire. Is this normal, and is there any treatment I can do at home to relieve the burning sensation? A salve, ointment, etc? I've been prescribed dilaudid 2mg but am reluctant to take them unless the pain is severe and unbearable. Note: my foot has been elevated above my heart approximately 10-12 hours a day.


**Biological Sex: M** (The post mentions that the subject is a male.)

**Gender identity: M** (Automatically filled based on Biological Sex)

**Age: 55** (The post mentions that the subject is 55 years old.)

**Height: 6'3"** (The post mentions that the subject is 6'3".)

**Height Units: feet/in** (The height is given in feet and inches.)

**Weight: 275** (The post mentions that the subject weighs 275 lbs.)

**Weight Units: lbs** (The weight is given in pounds.)

**Race: White** (The post mentions that the subject is Caucasian.)

**Diagnosis Based: True** (The post mentions a specific diagnosis of a shattered heel bone.)

**Symptom Based: True** (The post is centered on symptoms experienced by the subject, such as swelling and burning sensation.)

**Treatment Based: True** (The post asks for advice on home treatments to relieve the burning sensation.)

**Proxy Relationship: NA** (The post is written by the subject themselves, not on behalf of someone else.)

**Chronic: False** (The post mentions an injury that occurred recently, not something persistent or long-lasting.)

**Healthcare Consultation Status: In Consultation** (The subject has consulted an orthopedic surgeon and has surgery scheduled, but is seeking additional advice regarding symptoms.)

**Topic: Accident** (The post focuses on an injury to the heel bone)
