# Supplementary material for: Assessing Large Language Models in Building a Structured Dataset From AskDocs Subreddit Data: Methodological Study
Source: J Med Internet Res. 2025 Oct 22;27:e74094. doi: 10.2196/74094 (PMC12543290; doi:10.2196/74094)
Supplement: Multimedia Appendix 2 [file jmir-v27-e74094-s002.docx]

Multimedia Appendix 2 – Example Prompt for LLM

Use the following medical posts from r/askdocs as examples to fill in JSON fields for the final post.

JSON Fields:

Biological Sex (M, F, Unknown, NA): The biological sex of the subject.

Gender identity (M, F, Other, NA): The gender identity of the subject.

Age (Number, Unknown): The age of the subject (in years).

Height (e.g., 6'0, 170, Unknown): The height of the subject.

Height Units (feet/in, cm, m, NA.): The units in which the subject’s height is given. (Can be assumed by context)

Weight (Number, Unknown): The weight of the subject.

Weight Units (lbs, kg, NA): The units in which the subject’s weight is given. (Can be assumed by context)

Race (White, Asian, Black, Hispanic, Other, Unknown): The racial background of the subject.

Diagnosis Based (True, False, NA): Indicates if the post is focused on understanding or discussing a specific diagnosis.

Symptom Based (True, False, NA): Indicates if the post is centered on symptoms experienced by the subject without a known cause.

Treatment Based (True, False, NA): Indicates if the post is about a particular medication or treatment.

Proxy Relationship (NA, OP‚ Significant Other, OP‚ Friend, OP’s Child, etc., Other): If this post is on behalf of another person, this field should include the relationship that the subject (the one for whom the question is being asked) has with the Original Poster (the author of the post). If the post is not on behalf of another person, it should be marked NA.

Chronic (True, False, NA): Indicates if the medical condition mentioned in the post is chronic, meaning it is persistent or long-lasting.

Healthcare Consultation Status (Pre-Consultation, In Consultation, Post-Consultation, NA): Indicates the subject's stage in the healthcare consultation process at the time the post was made.

Topic (Anxiety, Back pain, Depression, Drugs or Alcohol, Earache, Headache, Hypertension, IBS, Rash, Sexual Health, Respiratory Infection, UTI, Vomiting/Diarrhea, Suicidality, Mental Health Other, Accident, Chest pain, Shortness of breath, Abdominal pain, Other): This category indicates the primary reason or focus of the post (from list of common medical topics). Select one or more topics that directly relate to the user's main concern or query. It is important to focus on the central issue the user is posting about, rather than unrelated elements of their medical history. Multiple topics can be selected if the post addresses more than one primary concern.

Example 1:

Title: Sudden buldgung vein on right side of forehead

Selftext: Im 26F, 7 months postpartum, 157cm height, 48kg. Had preeclampsia and emergency c-section at 36 weeks pregnant. Since then had some troubles with anxiety and high diastolic bp, (systolic always normal, below 120, diastolic now around 90 or higher, it was under 90 until recently, had some stress and anxiety) not on meds since my cardiologist said to try regulate it by sleeping more an less stress. So anyway yesterday I noticed a buldging vein on right side of my forehead, I had some pain days before when touching my right temple but figured it was cause I wasnt wearing my retainer I got because of some clicking jaw problems and pain so I started wearing it again and the pain stopped but the vein appeared (pic in the comments). Im wondering what could be causing this buldging vein and hoe to get rid of it???

I have had some significant weight loss after pregnancy cause Im breastfeeding and my son is alergic to a bunch of stuff so I was eating less stuff then before but there was no vain until yesterday. Im going to my doctor in a few days for something else (I wanna check my blood) so Im wondering if this is some kind of emergency, should I go sooner?

JSON Output:

{

"biological_sex": "Female",

"gender_identity": "Female",

"age": "26",

"height": "157",

"height_units": "cm",

"weight": "48",

"weight_units": "kg",

"race": "Unknown",

"diagnosis_based": false,

"symptom_based": true,

"treatment_based": false,

"proxy_relationship": "NA",

"chronic": false,

"healthcare_consultation_status": "Pre-Consultation",

"topic": ["Anxiety", "Other"]

}

Example 2:

Title: Two year old heart rate

Selftext: Hello! I have a 2 year old, 36’’ tall 28 lbs.

They started with a mild cold two-three days ago. Only real symptom is runny/stuffy nose. No fever, been drinking & eating somewhat normally.

I put our owlet sock on them because last time they had a cold they wound up with a nasty croup cough and trouble breathing so we went to the ER.

I noticed their heart rate is 120s-130s while sleeping. Normally it’s around 90.

I know with a fever usually heart rate is elevated but I’m concerned about it as there are no other symptoms. Is this concerning? What should I do?

They did have a staph infection according to urgent care two weeks ago. We finished a week of Cephalexin for it.

Thank you.

JSON Output:

{

"biological_sex": "Unknown",

"gender_identity": "Unknown",

"age": "2",

"height": "36",

"height_units": "in",

"weight": "28",

"weight_units": "lbs",

"race": "Unknown",

"diagnosis_based": true,

"symptom_based": true,

"treatment_based": false,

"proxy_relationship": "OP’s Child",

"chronic": false,

"healthcare_consultation_status": "Post-Consultation",

"topic": ["Respiratory Infection"]

}

Example 3:

Title: Does this sound like an ear infection?

Selftext: Age - 46 Sex - F Height - 5'8" Weight - 150 pounds - 11 stone Race - White Duration of complaint - a few days Location (Geographic and on body) - UK and ear Any existing relevant medical issues (if any) - nothing relevant Current medications (if any) - none

I'm very nervous about my ears because I used to get ear infections a lot. I suffered from swimmer's ear last summer but this is new.

A few days ago I woke up after a painful night's sleep with a slight headache and a wooshing in my left ear that comes and goes. It wooshes with my heartbeat. It will be very loud for about twenty seconds, then I can't hear it for about five seconds, and then it comes back for twenty seconds. It seems worse when I lay on my left side when sleeping.

I don't feel any pressure or trouble with my balance, and I don't have a fever. I haven't been ill lately. But I have had a mild headache for a few days and - I may be imagining it - I've had small prickles of pain in my inner ear.

Should I see my GP or wait to see if it gets worse?

JSON Output:

{

"biological_sex": "Female",

"gender_identity": "Female",

"age": "46",

"height": "5ft8in",

"height_units": "ft/in",

"weight": "150",

"weight_units": "lbs",

"race": "White",

"diagnosis_based": false,

"symptom_based": true,

"treatment_based": false,

"proxy_relationship": "NA",

"chronic": false,

"healthcare_consultation_status": "Pre-Consultation",

"topic": ["Earache", "Other"]

}

Example 4:

Title: Got rear-ended. ER said no problem, but cant stop throwing up?

Selftext: My friend got rear-ended on the highway. The car was totaled.

The ER sent him home, saying if he has no pain, then he must be fine. Few days later, his GP says the same thing.

1 week later, he's back at work. After swinging a hammer for 5 minutes, he starts throwing up.

Keeps happening like this consistently.

He's not fine right? What in the world is wrong?

He still has no pain. The only other thing he can notice, is when the vomit comes on, he suddenly starts feeling extremely fatigued, and the fatigue doesnt go away until he wakes up the next morning.

He cant puke everytime he swings a hammer. This is his livelihood. Its the only thing he knows.

What kind of specialty doctor does he need to see, to fix this?

Age: 45 Sex: M Height: 5'10 Weight: 165 Duration of complaint: 1 week Any existing relevant medical issues: none Current medications: none

JSON Output:

{

"biological_sex": "Male",

"gender_identity": "Male",

"age": "45",

"height": "5ft10in",

"height_units": "ft/in",

"weight": "165",

"weight_units": "lbs",

"race": "Unknown",

"diagnosis_based": false,

"symptom_based": true,

"treatment_based": false,

"proxy_relationship": "OP’s Friend",

"chronic": false,

"healthcare_consultation_status": "Post-Consultation",

"topic": ["Vomiting/Diarrhea", "Accident", "Other"]

}

Example 5:

Title: My gut's been a mess since my 6 year old decided to use me as a trampoline

Selftext: 38, female, white, no drugs/drinking/smoking, 137lbs and 5'4. CKD, no medication.

I was sleeping and my kiddo ran into the room, leapt on the bed, then jumped and landed with his butt right on my stomach. He's about 50lbs I'd say. Woke up like I'd been punched in the gut. This was 2 days ago. Yesterday I felt this weird intense bloated sensation, almost like when really constipated. Was a bit hard to eat anything because I kept feeling nauseated. Figured my guts were just sore.

Today is a little better but it still hurts an awful lot and I still have that weird bloated feeling.

I realize the general consensus is usually "just go see a doctor" about things, but we're poor folk and don't have insurance, and I owe like 10k already in medical bills from the latest kidney stone removal/surgery (this was like a year ago).

So I'd like to know what I should be looking out for because I really only want to go if I absolutely really need to. Just what to keep an eye on if anything.

Thank you much, appreciate what you do r/askdocs.

Edit: Guys... I appreciate what you're trying to do but I'm not asking for financial advice or anything like that. I understand what systems are and are not available to me. I really do.

JSON Output:

{

"biological_sex": "Female",

"gender_identity": "Female",

"age": "38",

"height": "5ft4in",

"height_units": "ft/in",

"weight": "137",

"weight_units": "lbs",

"race": "White",

"diagnosis_based": false,

"symptom_based": true,

"treatment_based": false,

"proxy_relationship": "NA",

"chronic": true,

"healthcare_consultation_status": "Pre-Consultation",

"topic": ["Abdominal pain", "Other"]

}

Example 6:

Title: My boyfriend’s heart issue?

Selftext: Hey guys! My boyfriend (22M, physically fit, good diet, doesn’t smoke or drink) has had an undiagnosed heart issue for the past four years.

He describes it as a kind of painful pressure in his chest that sometimes spreads to his shoulders and neck. Sometimes it’s random, but it often happens when he’s under emotional or physical stress, and once after eating a steak. Sometimes it happens every day, and other times he goes a week or so without a flare up.

He's been to a cardiologist who had absolutely no idea what was wrong. Scans seemed to show no physical abnormalities, and he was put on beta blockers for a time which he said helped somewhat. He has since stopped taking them.

I’m constantly worried about him, and I’m terrified that something happens to him and I won’t be able to help. He’s being really stubborn about seeing a new cardiologist for a second opinion, and I know he gets overwhelmed by my worry so that’s why I’m coming here for help.

Please let me know if you need any additional info? Thanks so much in advance!!

JSON Output:

{

"biological_sex": "Male",

"gender_identity": "Male",

"age": "22",

"height": "Unknown",

"height_units": "NA",

"weight": "Unknown",

"weight_units": "NA",

"race": "Unknown",

"diagnosis_based": false,

"symptom_based": true,

"treatment_based": true,

"proxy_relationship": "OP‚ÄôS Significant Other",

"chronic": true,

"healthcare_consultation_status": "Post-Consultation",

"topic": ["Chest pain"]

}

Example 7:

Title: [30M] A can of beer almost every night

Selftext: I'm an Asian male 30 years of age with a weight of 65kg and a height of 167cm, residing in Taiwan. Currently, I'm not suffering from any conditions, last year I just made a blood test out of curiosity for my health and the results turned out pretty normal except for my cholesterol which is in the normal range but closer to the ceiling. The doctor recommended me just to watch out what I eat but stated that I'm still young so nothing to worry about.

I have been drinking beer almost every night for the last 1 1/2 years. Typically five days a week, but there are some periods where it is a full seven days. I drink a can of 330 ml beer with 3-5% alcohol content usually, but there are infrequent times when it is two cans, but that is it.

My question is, will this habit be dangerous? Of course, as much as possible we want to eliminate this habit, but given that it is there, would it cause immediate danger? I plan to slowly change this habit, probably a decrease in frequency, but as you know, it's a habit.

JSON Output:

{

"biological_sex": "Male",

"gender_identity": "Male",

"age": "30",

"height": "167",

"height_units": "cm",

"weight": "65",

"weight_units": "kg",

"race": "Asian",

"diagnosis_based": false,

"symptom_based": false,

"treatment_based": false,

"proxy_relationship": "NA",

"chronic": false,

"healthcare_consultation_status": "Post-Consultation",

"topic": ["Drugs or Alcohol"]

}
